# Supplementary material for: Descending GABAergic pathway links brain sugar-sensing to peripheral nociceptive gating in Drosophila
Source: Nat Commun. 2023 Oct 16;14:6515. doi: 10.1038/s41467-023-42202-9 (PMC10579361; doi:10.1038/s41467-023-42202-9)
Supplement: Supplementary file 1 — Supplementary Information [file 41467_2023_42202_MOESM1_ESM.pdf]

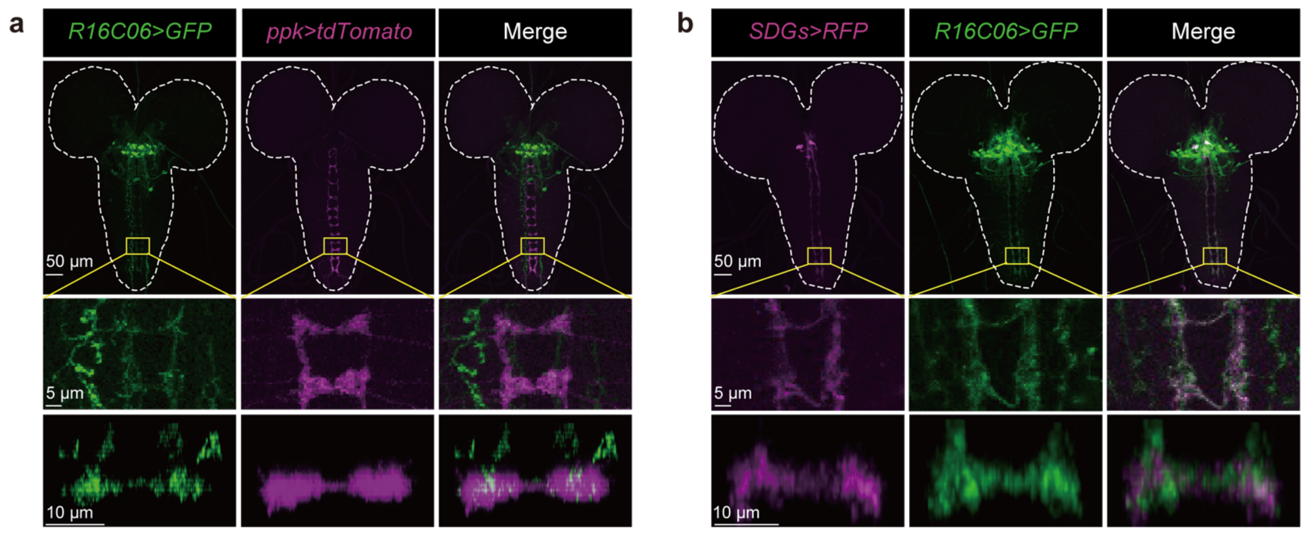

Supplementary Figure 1.

a Dual detection of *R16C06-GAL4*-labaled cells expressing mCD8::GFP (green) and C4da visualized by tdTomato (magenta). Top panels show the signals in the larval brain and VNC. Middle panels show magnified images of the abdominal A6–7 segments (the yellow squared area in the top panels) in the VNC. Bottom panels show transverse sections of the A7 segment. **b** Expression patterns of SDGs (magenta) overlapping with *R16C06-LexA*-labeled subpopulations (green). See Supplementary Table 1 for full genotypes.

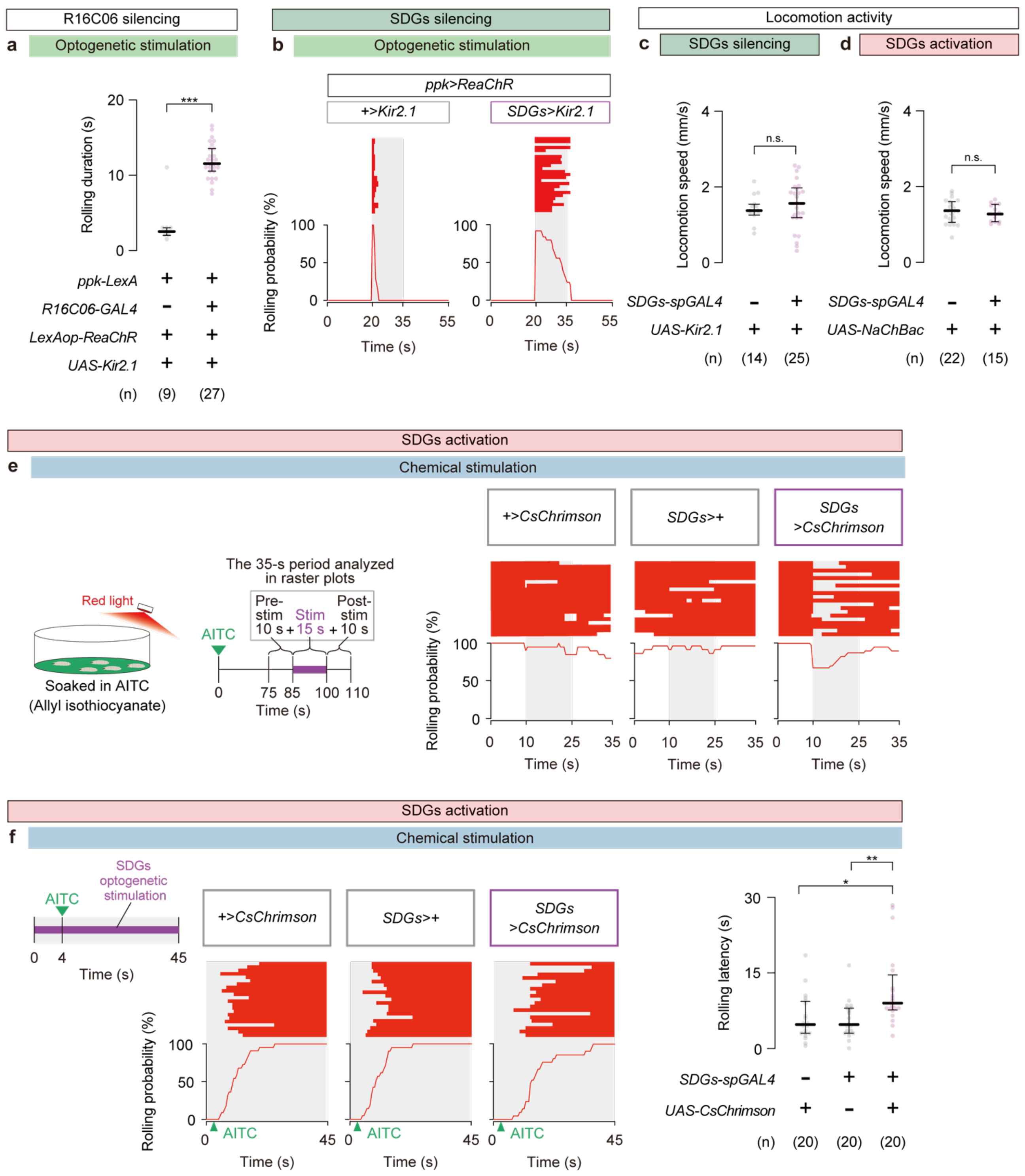

Supplementary Figure 2.

a Rolling duration in larvae expressing ReaChR in C4da. Larvae were activated by a red-light LED at 35 µW/mm^2 while *R16C06-GAL4* neurons were genetically silenced. In this and following panels, ‘n’ indicates the number of biologically independent animals used for each group, and the thick line and the

thin error bar represent the median and interquartile range, respectively. *** $p < 0.0005$ (Mann–Whitney U-test). **b** Top, raster plots of rolling (red) and non-rolling (blank) states under the optogenetic stimulation of C4da (grey-shaded box). Each row represents one tested larva. Bottom, rolling probability at each time point. **c**, **d** Locomotion activity in larvae expressing either Kir2.1 (**c**) or NaChBac (**d**) in SDGs to manipulate neural activities, respectively. Note that locomotion speed of Kir2.1-expressing larvae was measured from the pre-stimulation period of Fig. 2a. n.s. $p \geq 0.05$ (Mann–Whitney U-test). **e** Left and middle, a schematic design and the time course of SDGs optogenetic activation on AITC-elicited rolling. Note that raster plots were drawn from the 35-s observation period consisting of 10 s pre-stimulation (no LED) + 15 s stimulation with LED + 10 s post-stimulation (no LED). Right, raster plots and line graphs showing the rolling probability at each time point in response to AITC. **f** Effect of SDGs optogenetic activation on AITC-elicited rolling initiation. Left, time course of SDGs optogenetic activation and AITC treatment. Note that SDGs were activated prior to AITC application, which is different from Supplementary Fig. 2e. Middle, raster plots showing the rolling probability at each time point in response to AITC. Right, latency of the rolling onset after the AITC application. ** $p < 0.005$, * $p < 0.05$ (Mann–Whitney U-test with Bonferroni correction). See Supplementary Table 1 for full genotypes. Source data are provided as a Source Data file.

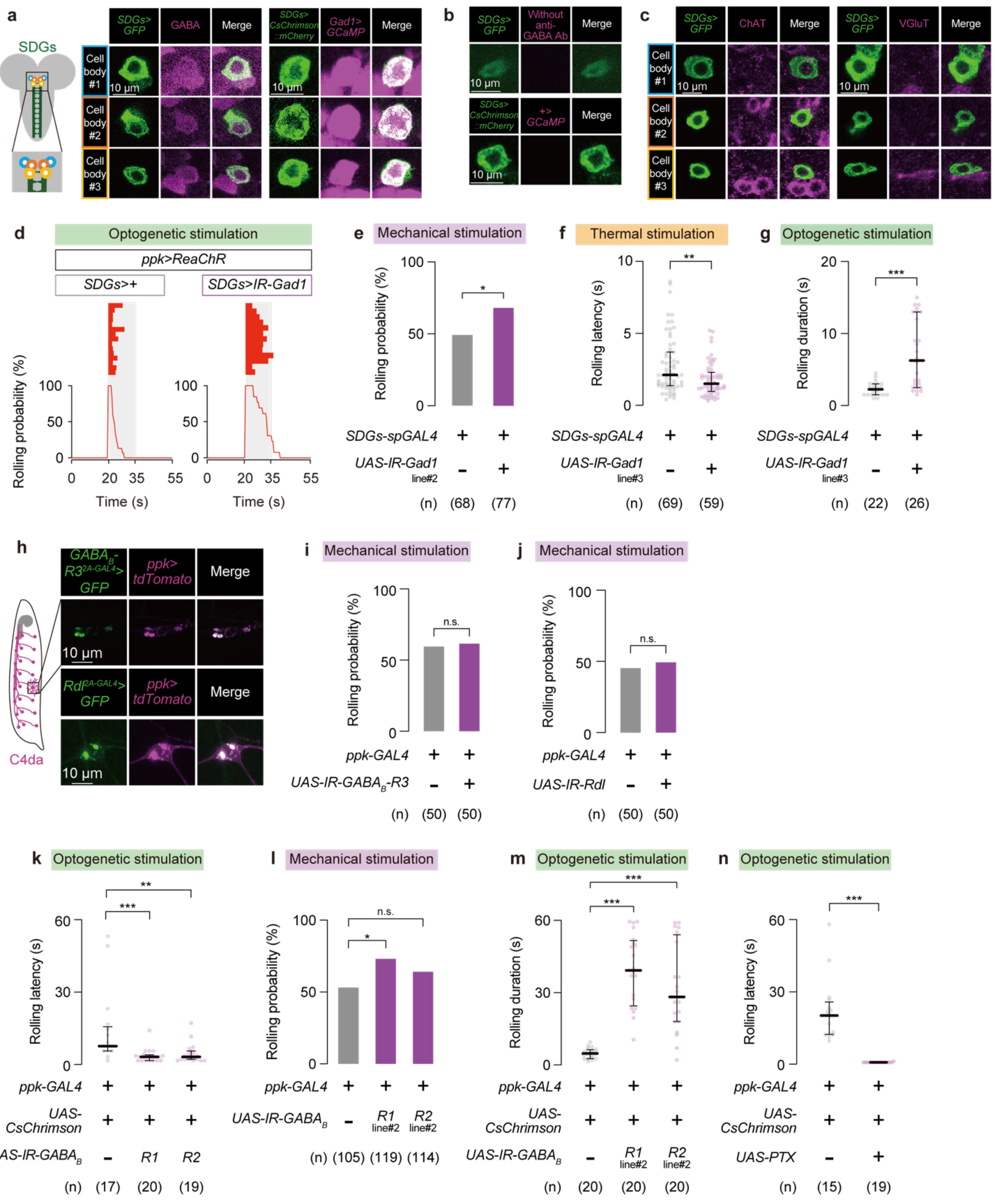

Supplementary Figure 3.

a Left, immunostaining of GABA (magenta) at the cell bodies of SDGs expressing mCD8::GFP (green). Right, dual labeling of SDGs with CsChrimson::mCherry and *Gad1-LexA*-driven GCaMP. **b** Negative controls for GABA and *Gad1-LexA*-driven GCaMP staining at the cell bodies of SDGs. Top,

immunostaining of GABA without adding the primary antibody (magenta). Bottom, "no-*LexA*" control larvae lacking *Gad1-LexA*. **c** Immunostaining of either choline acetyltransferase (ChAT, magenta in the left 3 × 3 panels) or vesicular glutamate transporter (VGluT, magenta in the right 3 × 3 panels), respectively, at the cell bodies of SDGs expressing mCD8::GFP (green). **d** Rolling (red) or non-rolling (blank) states and rolling probability under *Gad1* knockdown in SDGs. Rolling was elicited by optogenetic stimulation of C4da for 15 s (grey-shaded box). Each row in the raster plots represents one tested larva. **e–g** Nociceptive suppression via *Gad1* knockdown in SDGs using additional RNAi lines. Larvae were assessed for rolling probability by mechanical stimulation (**e**), rolling latency after noxious heat application (**f**), and duration of rolling triggered by C4da optogenetic stimulation (**g**). *UAS-IR-Gad1* line #2, *TRiP.JF02916* in attP2 (BL#28079); line #3, *GD8508* (VDRC#32344). In this and following panels, 'n' indicates the number of biologically independent animals used for each group, and the thick line and the thin error bar represent the median and interquartile range, respectively. *** $p < 0.0005$, ** $p < 0.005$, * $p < 0.05$ (Mann–Whitney U-test). **h** Colocalization of GFP driven by either *GABA*$_B$*-R3* or *Rdl* (green) with *ppk-CD4-tdTomato* (magenta) in C4da cell bodies. **i**, **j** Behavioral phenotypes under *GABA*$_B$*-R3* (**i**) and *Rdl* (**j**) knockdown in C4da. C4da was activated by the mechanical stimulation. n.s. $p \geq 0.05$ (Fisher's exact test). **k** Effect of *GABA*$_B$*-R*s knockdown on the rolling latency after C4da optogenetic stimulation. *** $p < 0.0005$, ** $p < 0.005$ (Mann–Whitney U-test with Bonferroni correction). **l**, **m** Nociceptive suppression via *GABA*$_B$*-Rs* knockdown in C4da using additional RNAi lines. Larvae were assessed for rolling probability by mechanical stimulation (**l**) and duration of rolling triggered by C4da optogenetic stimulation (**m**). *UAS-IR-GABA*$_B$*-R1* line #2, *KK109166* in VIE260b (VDRC#101440); *UAS-IR-GABA*$_B$*-R2* line #2, *KK100020* in VIE260b (VDRC#110268). *** $p < 0.0005$, * $p < 0.05$, n.s. $p \geq 0.05$ (Mann–Whitney U-test with Bonferroni correction). **n** Effect of PTX expression on the rolling latency after C4da optogenetic stimulation. *** $p < 0.0005$ (Mann–Whitney U-test). See Supplementary Table 1 for full genotypes. Source data are provided as a Source Data file.

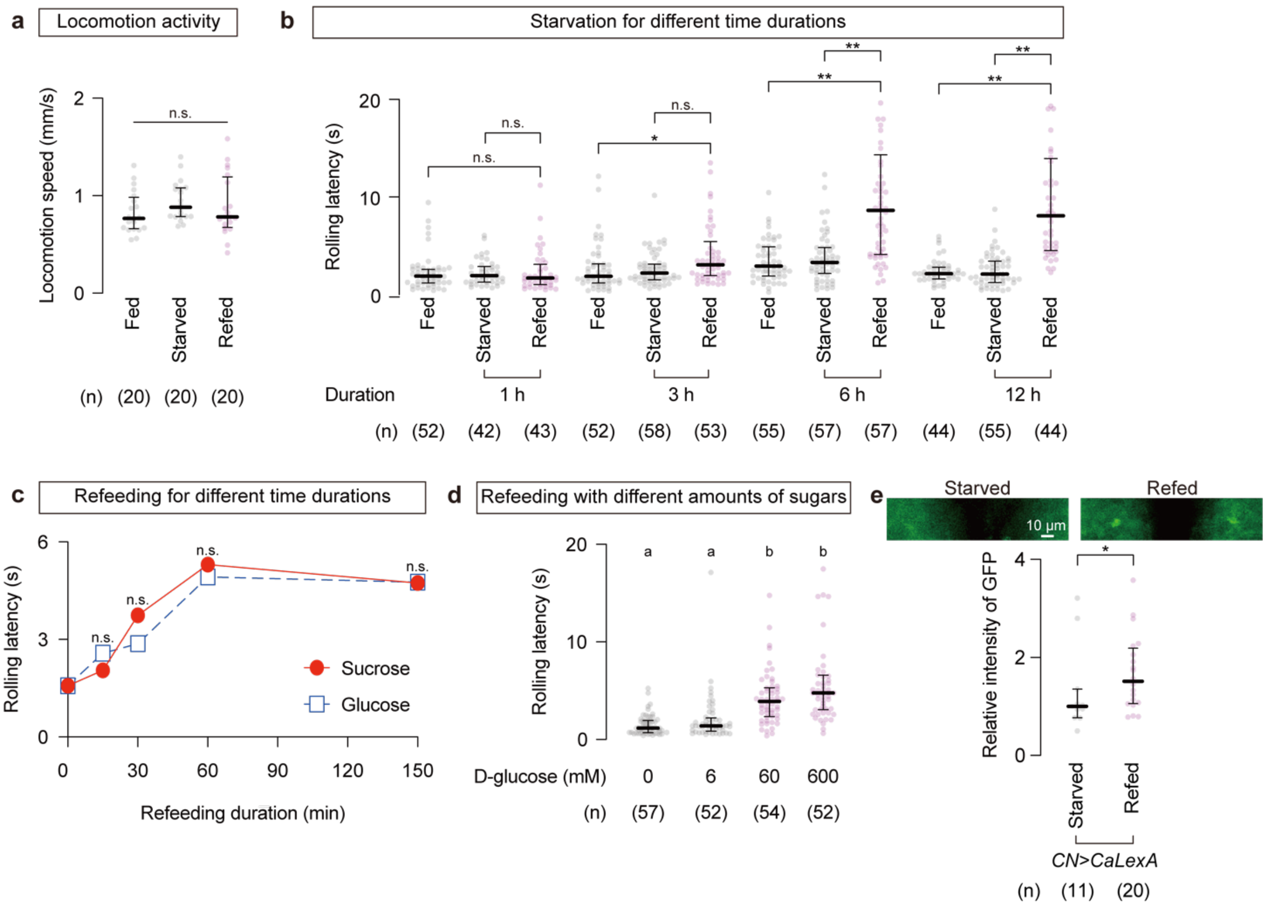

Supplementary Figure 4.

a Larval locomotion activity under different nutritive states. In this and following panels except for (**e**), ‘n’ indicates the number of biologically independent animals used for each group, and the thick line and the thin error bar represent the median and interquartile range, respectively. n.s. $p \geq 0.05$ (Kruskal–Wallis one-way ANOVA). **b** Latency of the rolling onset in larvae starved for different time durations. ** $p < 0.005$, * $p < 0.05$, n.s. $p \geq 0.05$ (Mann–Whitney U-test with Bonferroni correction). **c** Relationship between refeeding duration and rolling latency. The horizontal axis indicates the time spent for refeeding larvae with either sucrose (red-filled circles) or D-glucose (blue-opened squares). n.s. $p \geq 0.05$ (Mann–Whitney U-test with Bonferroni correction). **d** Dose-dependent effect of D-glucose refeeding on larval rolling latency. a–b $p < 0.0001$ (Kruskal–Wallis one-way ANOVA and Dunn’s multiple comparison test). **e** Effect of sugar refeeding on CN neuronal activity visualized by CaLexA. Top, representative images showing cell bodies of CN neurons labeled by the CaLexA reporter. Bottom, GFP signal

intensities quantified at the cell bodies, normalized to the median value of the starved group. The thick line and the thin error bar represent the median and interquartile range, respectively. Note that ‘n’ indicates the number of cell bodies analyzed for each condition. * $p < 0.05$ (Mann–Whitney U-test). See Supplementary Table 1 for full genotypes. Source data are provided as a Source Data file.

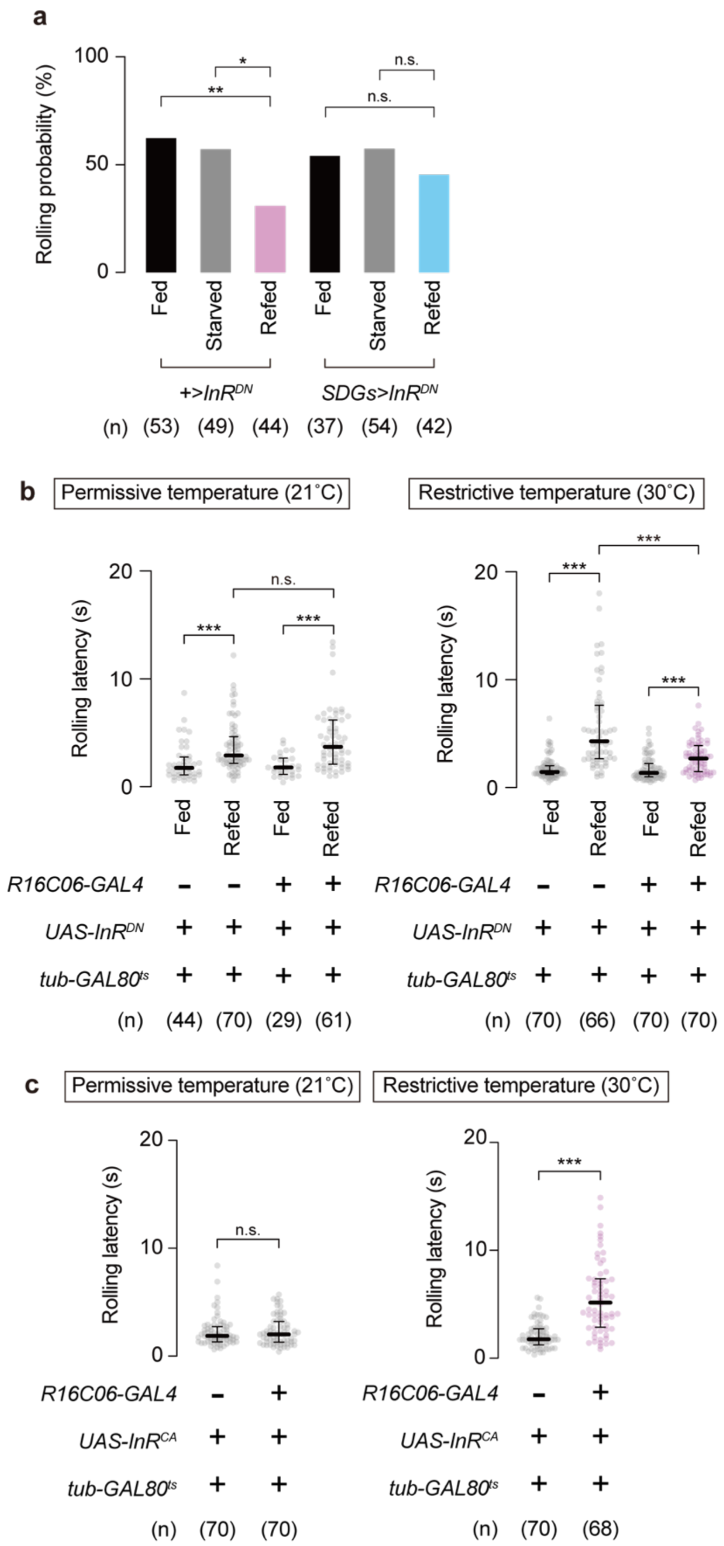

Supplementary Figure 5.

a Mechano-nociceptive responses in larvae with InR function impaired specifically in SDGs. In this and following panels, ‘n’ indicates the number of biologically independent animals used for each group. ** $p < 0.005$, * $p < 0.05$, n.s. $p \geq 0.05$ (Fisher’s exact test with Bonferroni correction). **b** Effect of temporal

InR inactivation on thermo-nociceptive rolling latency. Rearing temperature was either kept constant at 21˚C (left, "permissive temperature" for $GAL80^{ts}$) or shifted from 21˚C to 30˚C during the 6-h starvation followed by 1 h of sugar refeeding (right, "restrictive temperature" for $GAL80^{ts}$). InR^{DN}, the dominant negative form of InR. The thick line and the thin error bar represent the median and interquartile range, respectively. *** $p < 0.0005$, n.s. $p \geq 0.05$ (Mann–Whitney U-test with Bonferroni correction). **c** Effect of conditional InR activation on thermo-nociceptive rolling latency. Rearing temperature was either kept constant at 21˚C (left, "permissive temperature" for $GAL80^{ts}$) or shifted to 30˚C for the last 15 h preceding the heat assay (right, "restrictive temperature" for $GAL80^{ts}$). InR^{CA}, the constitutively activated variant of InR. *** $p < 0.0005$, n.s. $p \geq 0.05$ (Mann–Whitney U-test). See Supplementary Table 1 for full genotypes. Source data are provided as a Source Data file.

Supplementary Table 1. Full genotypes of flies used in this study

Figure panel	*Abbreviated genotype*	Complete genotype
Fig. 1b		w; R21F01-p65.AD (attP40)/+; R93B07-GAL4.DBD (attP2)/UAS-CD4-tdTomato (VK00033), UAS-Stinger
Fig. 1c		w; R21F01-p65.AD (attP40)/ppk-CD4-tdTomato; R93B07-GAL4.DBD (attP2)/10XUAS-mCD8::GFP (attP2)
Fig. 1d		w; R21F01-p65.AD (attP40)/UAS-brpD3::mCherry; R93B07-GAL4.DBD (attP2)/20XUAS-IVS-mCD8::GFP (attP2)
Fig. 1e		w; R21F01-p65.AD (attP40), 20XUAS-CsChrimson::mCherry (su(Hw)attP5)/UAS-nsyb-$spGFP_{1-10}$, LexAop-CD4-spGFP11; R93B07-GAL4.DBD (attP2)/ppk-nlsLexA::p65 (attP2)
Supplementary Fig. 1a		w; +/ppk-CD4-tdTomato; R16C06-GAL4 (attP2)/10XUAS-mCD8::GFP (attP2)
Supplementary Fig. 1b		y^1 w^* 10XUAS-mCD8::RFP (attP18), 13XLexAop2-mCD8::GFP (su(Hw)attP8)/w or Y; R21F01-p65.AD (attP40)/R16C06-LexA (attP40); R93B07-GAL4.DBD (attP2)/+
Fig. 2a	ppk>ReaChR; +>Kir2.1	w; +/UAS-Kir2.1::EGFP; +/ppk-nlsLexA::p65 (attP2), LexAop-ReaChR (VK00005)
	ppk>ReaChR; SDGs>+	w; R21F01-p65.AD (attP40)/+; R93B07-GAL4.DBD (attP2)/ppk-nlsLexA::p65 (attP2), LexAop-ReaChR (VK00005)
	ppk>ReaChR; SDGs>Kir2.1	w; R21F01-p65.AD (attP40)/UAS-Kir2.1::EGFP; R93B07-GAL4.DBD (attP2)/ppk-nlsLexA::p65 (attP2), LexAop-ReaChR (VK00005)
Fig. 2b	ppk>ReaChR; SDGs>+	w; R21F01-p65.AD (attP40)/+; R93B07-GAL4.DBD (attP2)/ppk-nlsLexA::p65 (attP2), LexAop-ReaChR (VK00005)
	ppk>ReaChR; SDGs>Kir2.1	w; R21F01-p65.AD (attP40)/UAS-Kir2.1::EGFP; R93B07-GAL4.DBD (attP2)/ppk-nlsLexA::p65 (attP2), LexAop-ReaChR (VK00005)
Fig. 2c	+>Kir2.1	w; +/UAS-Kir2.1::EGFP; +
	SDGs>+	w; +/R21F01-p65.AD (attP40); +/R93B07-GAL4.DBD (attP2)
	SDGs>Kir2.1	w; R21F01-p65.AD (attP40)/UAS-Kir2.1::EGFP; +/R93B07-GAL4.DBD (attP2)
Fig. 2d	+>Kir2.1	w; +/UAS-Kir2.1::EGFP; +
	SDGs>+	w; +/R21F01-p65.AD (attP40); +/R93B07-GAL4.DBD (attP2)
	SDGs>Kir2.1	w; R21F01-p65.AD (attP40)/UAS-Kir2.1::EGFP; +/R93B07-GAL4.DBD (attP2)

Fig. 2e	+>GtACR1	w; +; UAS-GtACR1::EYFP (attP2)/+
	SDGs>+	w; +/R21F01-p65.AD (attP40); +/R93B07-GAL4.DBD (attP2)
	SDGs>GtACR1	w; R21F01-p65.AD (attP40)/+; UAS-GtACR1::EYFP (attP2)/R93B07-GAL4.DBD (attP2)
Fig. 2f	+>NaChBac	w; +/UAS-NaChBac::EGFP; +
	SDGs>+	w; +/R21F01-p65.AD (attP40); +/R93B07-GAL4.DBD (attP2)
	SDGs>NaChBac	w; R21F01-p65.AD (attP40)/UAS-NaChBac::EGFP; +/R93B07-GAL4.DBD (attP2)
Fig. 2g	+>CsChrimson	w; +/20XUAS-CsChrimson::mCherry (su(Hw)attP5); +
	SDGs>+	w; +/R21F01-p65.AD (attP40); +/R93B07-GAL4.DBD (attP2)
	SDGs>CsChrimson	w; R21F01-p65.AD (attP40)/20XUAS-CsChrimson::mCherry (su(Hw)attP5); +/R93B07-GAL4.DBD (attP2)
Fig. 2h	+>CsChrimson	w; +/20XUAS-CsChrimson::mCherry (su(Hw)attP5); +
	SDGs>+	w; +/R21F01-p65.AD (attP40); +/R93B07-GAL4.DBD (attP2)
	SDGs>CsChrimson	w; R21F01-p65.AD (attP40)/20XUAS-CsChrimson::mCherry (su(Hw)attP5); +/R93B07-GAL4.DBD (attP2)
Supplementary Fig. 2a	ppk>ReaChR; +>Kir2.1	w; +/UAS-Kir2.1::EGFP; +/ppk-nls::LexA::p65 (attP2), LexAop-ReaChR (VK00005)
	ppk>ReaChR; R16C06>Kir2.1	w; +/UAS-Kir2.1::EGFP; R16C06-GAL4 (attP2)/ppk-nlsLexA::p65 (attP2), LexAop-ReaChR (VK00005)
Supplementary Fig. 2b	ppk>ReaChR; +>Kir2.1	w; +/UAS-Kir2.1::EGFP; +/ppk-nlsLexA::p65 (attP2), LexAop-ReaChR (VK00005)
	ppk>ReaChR; SDGs>Kir2.1	w; R21F01-p65.AD (attP40)/UAS-Kir2.1::EGFP; R93B07-GAL4.DBD (attP2)/ppk-nlsLexA::p65 (attP2), LexAop-ReaChR (VK00005)
Supplementary Fig. 2c	+>Kir2.1	w; +/UAS-Kir2.1::EGFP; +
	SDGs>Kir2.1	w; R21F01-p65.AD (attP40)/UAS-Kir2.1::EGFP; +/R93B07-GAL4.DBD (attP2)
Supplementary Fig. 2d	+>NaChBac	w; +/UAS-NaChBac::EGFP; +
	SDGs>NaChBac	w; R21F01-p65.AD (attP40)/UAS-NaChBac::EGFP; +/R93B07-GAL4.DBD (attP2)
Supplementary	+>CsChrimson	w; +/20XUAS-CsChrimson::mCherry (su(Hw)attP5); +

Fig. 2e	SDGs>+	w; +/R21F01-p65.AD (attP40); +/R93B07-GAL4.DBD (attP2)
	SDGs>CsChrimson	w; R21F01-p65.AD (attP40)/20XUAS-CsChrimson::mCherry (su(Hw)attP5); +/R93B07-GAL4.DBD (attP2)
Supplementary Fig. 2f	+>CsChrimson	w; +/20XUAS-CsChrimson::mCherry (su(Hw)attP5); +
	SDGs>+	w; +/R21F01-p65.AD (attP40); +/R93B07-GAL4.DBD (attP2)
	SDGs>CsChrimson	w; R21F01-p65.AD (attP40)/20XUAS-CsChrimson::mCherry (su(Hw)attP5); +/R93B07-GAL4.DBD (attP2)
Fig. 3a	SDGs>GFP	w; R21F01-p65.AD (attP40)/+; R93B07-GAL4.DBD (attP2)/20XUAS-IVS-mCD8::GFP (attP2)
	SDGs>CsChrimson::mCherry	w; R21F01-p65.AD (attP40)/20XUAS-CsChrimson::mCherry (su(Hw)attP5); R93B07-GAL4.DBD (attP2), 13XLexAop-IVS-jGCaMP7s (VK00005)/Gad1-Trojan-LexA-QFAD
Fig. 3b	SDGs>+	w (derived from w^{1118}); R21F01-p65.AD (attP40)/CaryP (attP40); + (derived from w^{1118})/R93B07-GAL4.DBD (attP2)
	SDGs>IR-Gad1	w (derived from w^{1118}); R21F01-p65.AD (attP40)/UAS-IR-Gad1 (TRiP.HMC03350) (attP40); + (derived from w^{1118})/R93B07-GAL4.DBD (attP2)
Fig. 3c	SDGs>+	w/y^1 v^1or Y; R21F01-p65.AD (attP40)/CaryP (attP40); +/R93B07-GAL4.DBD (attP2)
	SDGs>IR-Gad1	w/y^1 v^1or Y; R21F01-p65.AD (attP40)/UAS-IR-Gad1 (TRiP.HMC03350) (attP40); +/R93B07-GAL4.DBD (attP2)
Fig. 3d	ppk>ReaChR;<br>SDGs>+	w; R21F01-p65.AD (attP40)/CaryP (attP40); R93B07-GAL4.DBD (attP2)/ppk-nlsLexA::p65 (attP2), LexAop-ReaChR (VK00005)
	ppk>ReaChR;<br>SDGs>IR-Gad1	w; R21F01-p65.AD (attP40)/UAS-IR-Gad1 (TRiP.HMC03350) (attP40); R93B07-GAL4.DBD (attP2)/ppk-nlsLexA::p65 (attP2), LexAop-ReaChR (VK00005)
Fig. 3e	GABA$_B$-R1$^{2A\text{-}GAL4}$>GFP	w; GABA$_B$-R1$^{2A\text{-}GAL4}$/ppk-CD4-tdTomato; 10XUAS-mCD8::GFP (attP2)/ppk-CD4-tdTomato
	GABA$_B$-R2$^{2A\text{-}GAL4}$>GFP	w; ppk-CD4-tdTomato/ppk-CD4-tdTomato; GABA$_B$-R2$^{2A\text{-}GAL4}$/10XUAS-mCD8::GFP (attP2)
Fig. 3f	ppk>+	w, ppk-GAL4/Y; +; CaryP (attP2)
	ppk>IR-GABA$_B$-R1	w, ppk-GAL4/Y; +; UAS-IR-GABA$_B$-R1 (TRiP.HMC03388) (attP2)
	ppk>IR-GABA$_B$-R2	w, ppk-GAL4/Y; +; UAS-IR-GABA$_B$-R2 (TRiP.HMC02975) (attP2)
Fig. 3g	ppk>CsChrimson	w, ppk-GAL4/Y; +/20XUAS-CsChrimson::mCherry (su(Hw)attP5); CaryP (attP2)

	ppk>CsChrimson, IR-GABA$_B$*-R1*	*w, ppk-GAL4/Y; +/20XUAS-CsChrimson::mCherry* (su(Hw)attP5)*; UAS-IR-GABA*$_B$*-R1 (TRiP.HMC03388)* (attP2)
	ppk>CsChrimson IR-GABA$_B$*-R2*	*w, ppk-GAL4/Y; +/20XUAS-CsChrimson::mCherry* (su(Hw)attP5)*; UAS-IR-GABA*$_B$*-R2 (TRiP.HMC02975)* (attP2)
Fig. 3h	*ppk>CsChrimson*	*w, ppk-GAL4/Y; +/20XUAS-CsChrimson::mCherry* (su(Hw)attP5)*; +*
	ppk>CsChrimson, PTX	*w, ppk-GAL4/Y; +/20XUAS-CsChrimson::mCherry* (su(Hw)attP5)*; +/ry*^{506}*, UAS-PTX*
Supplementary Fig. 3a	*SDGs>GFP*	*w; R21F01-p65.AD* (attP40)*/+; R93B07-GAL4.DBD* (attP2)*/20XUAS-IVS-mCD8::GFP* (attP2)
	SDGs>CsChrimson::mCherry; *Gad1>GCaMP*	*w; R21F01-p65.AD* (attP40)*/20XUAS-CsChrimson::mCherry* (su(Hw)attP5)*; R93B07-GAL4.DBD* (attP2)*, 13XLexAop-IVS-jGCaMP7s* (VK00005)*/Gad1-Trojan-LexA-QFAD*
Supplementary Fig. 3b	*SDGs>GFP*	*w; R21F01-p65.AD* (attP40)*/+; R93B07-GAL4.DBD* (attP2)*/20XUAS-IVS-mCD8::GFP* (attP2)
	SDGs>CsChrimson::mCherry; *+>GCaMP*	*w; R21F01-p65.AD* (attP40)*/20XUAS-CsChrimson::mCherry* (su(Hw)attP5)*; R93B07-GAL4.DBD* (attP2)*, 13XLexAop-IVS-jGCaMP7s* (VK00005)/+
Supplementary Fig. 3c	*SDGs>GFP*	*w; R21F01-p65.AD* (attP40)*/+; R93B07-GAL4.DBD* (attP2)*/20XUAS-IVS-mCD8::GFP* (attP2)
Supplementary Fig. 3d	*ppk>ReaChR*; *SDGs>+*	*w; R21F01-p65.AD* (attP40)*/CaryP* (attP40)*; R93B07-GAL4.DBD* (attP2)*/ppk-nlsLexA::p65* (attP2)*, LexAop-ReaChR* (VK00005)
	ppk>ReaChR; *SDGs>IR-Gad1*	*w; R21F01-p65.AD* (attP40)*/UAS-IR-Gad1 (TRiP.HMC03350)* (attP40)*; R93B07-GAL4.DBD* (attP2)*/ppk-nlsLexA::p65* (attP2)*, LexAop-ReaChR* (VK00005)
Supplementary Fig. 3e	*SDGs>+*	*w/y*^{1} *v*^{1}or Y*; R21F01-p65.AD* (attP40)*/+; R93B07-GAL4.DBD* (attP2)*/CaryP* (attP2)
	SDGs>IR-Gad1 (line#2)	*w/y*^{1} *v*^{1}or Y*; R21F01-p65.AD* (attP40)*/+; R93B07-GAL4.DBD* (attP2)*/UAS-IR-Gad1 (TRiP.JF02916)* (attP2)
Supplementary Fig. 3f	*SDGs>+*	*w; R21F01-p65.AD* (attP40)*/GD control (GD60000); R93B07-GAL4.DBD* (attP2)*/UAS-Dcr-2*
	SDGs>IR-Gad1 (line#3)	*w; R21F01-p65.AD* (attP40)*/UAS-IR-Gad1 (GD8508); R93B07-GAL4.DBD* (attP2)*/UAS-Dcr-2*
Supplementary Fig. 3g	*ppk>ReaChR*; *SDGs>+*	*w; R21F01-p65.AD* (attP40)*/GD control (GD60000); R93B07-GAL4.DBD* (attP2)*/ppk-nlsLexA::p65* (attP2)*, LexAop-ReaChR* (VK00005)
	ppk>ReaChR; *SDGs>IR-Gad1* (line#3)	*w; R21F01-p65.AD* (attP40)*/UAS-IR-Gad1 (GD8508); R93B07-GAL4.DBD* (attP2)*/ppk-nlsLexA::p65* (attP2)*, LexAop-ReaChR* (VK00005)

Supplementary Fig. 3h	GABA$_B$-R3$^{2A\text{-}AD\text{-}GAL4}$>GFP; ppk>tdTomato	w; GABA$_B$-R3$^{2A\text{-}AD\text{-}GAL4}$/ppk-CD4-tdTomato; 10XUAS-mCD8::GFP (attP2)/ppk-CD4-tdTomato
	Rdl$^{2A\text{-}GAL4}$>GFP; ppk>tdTomato	w; ppk-CD4-tdTomato/ppk-CD4-tdTomato; Rdl$^{2A\text{-}GAL4}$/10XUAS-mCD8::GFP (attP2)
Supplementary Fig. 3i	ppk>+	w, ppk-GAL4/Y; CaryP (attP40)/+; +
	ppk>IR-GABA$_B$-R3	w, ppk-GAL4/Y; UAS-IR-GABA$_B$-R3 (TRiP.HMC02989) (attP40)/+; +
Supplementary Fig. 3j	ppk>+	w, ppk-GAL4/Y; CaryP (attP40); +
	ppk>IR-Rdl	w, ppk-GAL4/Y; UAS-IR-Rdl (TRiP.HMC03643) (attP40); +
Supplementary Fig. 3k	ppk>CsChrimson	w, ppk-GAL4/Y; +/20XUAS-CsChrimson::mCherry (su(Hw)attP5); CaryP (attP2)
	ppk>CsChrimson, IR-GABA$_B$-R1	w, ppk-GAL4/Y; +/20XUAS-CsChrimson::mCherry (su(Hw)attP5); UAS-IR-GABA$_B$-R1 (TRiP-HMC03388) (attP2)
	ppk>CsChrimson, IR-GABA$_B$-R2	w, ppk-GAL4/Y; +/20XUAS-CsChrimson::mCherry (su(Hw)attP5); UAS-IR-GABA$_B$-R2 (TRiP.HMC02975) (attP2)
Supplementary Fig. 3l	ppk>+	w, ppk-GAL4/Y; KK control (VDRC60100) (VIE260b)/+; UAS-Dcr-2/+
	ppk>IR-GABA$_B$-R1 (line#2)	w, ppk-GAL4/Y; UAS-IR-GABA$_B$-R1 (KK109166) (VIE260b)/+; UAS-Dcr-2/+
	ppk>IR-GABA$_B$-R2 (line#2)	w, ppk-GAL4/Y; UAS-IR-GABA$_B$-R2 (KK100020) (VIE260b)/+; UAS-Dcr-2/+
Supplementary Fig. 3m	ppk>CsChrimson	w, ppk-GAL4/Y; KK control (VDRC60100) (VIE260b)/20XUAS-CsChrimson::mCherry (su(Hw)attP5); +
	ppk>CsChrimson, IR-GABA$_B$-R1 (line#2)	w, ppk-GAL4/Y; UAS-IR-GABA$_B$-R1 (KK109166) (VIE260b)/20XUAS-CsChrimson::mCherry (su(Hw)attP5); UAS-Dcr-2/+
	ppk>CsChrimson, IR-GABA$_B$-R2 (line#2)	w, ppk-GAL4/Y; UAS-IR-GABA$_B$-R2 (KK100020) (VIE260b)/20XUAS-CsChrimson::mCherry (su(Hw)attP5); UAS-Dcr-2/+
Supplementary Fig. 3n	ppk>CsChrimson	w, ppk-GAL4/Y; +/20XUAS-CsChrimson::mCherry (su(Hw)attP5); +
	ppk>CsChrimson, PTX	w, ppk-GAL4/Y; +/20XUAS-CsChrimson::mCherry (su(Hw)attP5); +/ry^{506}, UAS-PTX
Fig. 4a	ppk>ChR2, RGECO; SDGs>+	w; R21F01-p65.AD (attP40)/13XLexAop2-IVS-NES-jRGECO1a-p10 (su(Hw)attP5); R93B07-GAL4.DBD (attP2)/ppk-nlsLexA::p65 (attP2), 13XLexAop2-ChR2.T159C-HA (VK00013)
	ppk>ChR2, RGECO; SDGs>Kir2.1	w; R21F01-p65.AD (attP40), UAS-Kir2.1::EGFP/13XLexAop2-IVS-NES-jRGECO1a-p10 (su(Hw)attP5); R93B07-GAL4.DBD (attP2)/ppk-nlsLexA::p65 (attP2), 13XLexAop2-ChR2.T159C-HA (VK00013)

Fig. 4b	ppk>ChR2, RGECO; SDGs>+	w; R21F01-p65.AD (attP40)/13XLexAop2-IVS-NES-jRGECO1a-p10 (su(Hw)attP5); R93B07-GAL4.DBD (attP2)/ppk-nlsLexA::p65 (attP2), 13XLexAop2-ChR2.T159C-HA (VK00013)
	ppk>ChR2, RGECO; SDGs>NaChBac	w; R21F01-p65.AD (attP40), UAS-NaChBac::EGFP/13XLexAop2-IVS-NES-jRGECO1a-p10 (su(Hw)attP5); R93B07-GAL4.DBD (attP2)/ppk-nlsLexA::p65 (attP2), 13XLexAop2-ChR2.T159C-HA (VK00013)
Fig. 4d	ppk>ChR2, RGECO	w; +/13XLexAop2-IVS-NES-jRGECO1a-p10 (su(Hw)attP5); +/ppk-nlsLexA::p65 (attP2), 13XLexAop2-ChR2.T159C-HA (VK00013)
Fig. 4e	ppk>ChR2, RGECO	w, ppk-GAL4/Y; 20XUAS-ChR2.T159C-HA (VK00018)/20XUAS-droRGECO; +/CaryP (attP2)
	ppk>ChR2, RGECO, IR-$GABA_B$-R1	w, ppk-GAL4/Y; 20XUAS-ChR2.T159C-HA (VK00018)/20XUAS-droRGECO; +/UAS-IR-$GABA_B$-R1 (TRiP.HMC03388) (attP2)
	ppk>ChR2, RGECO, IR-$GABA_B$-R2	w, ppk-GAL4/Y; 20XUAS-ChR2.T159C-HA (VK00018)/20XUAS-droRGECO; +/UAS-IR-$GABA_B$-R2 (TRiP.HMC02975) (attP2)
Fig. 4f	ppk>ChR2, RGECO	w, ppk-GAL4/Y; 20XUAS-ChR2.T159C-HA (VK00018)/20XUAS-droRGECO; +
	ppk>ChR2, RGECO, PTX	w, ppk-GAL4/Y; 20XUAS-ChR2.T159C-HA (VK00018)/20XUAS-droRGECO; +/ry^{506}, UAS-PTX
Fig. 4g	ppk>ChR2; SDGs>RGECO	w; R21F01-p65.AD (attP40)/20XUAS-droRGECO; R93B07-GAL4.DBD (attP2)/ppk-nlsLexA::p65 (attP2), 13XLexAop2-ChR2.T159C-HA (VK00013)
Fig. 5b		w^{1118}; +; +
Fig. 5c		w^{1118}; +; +
Fig. 5d		w; +; +/ppk-nlsLexA::p65 (attP2), LexAop-ReaChR (VK00005)
Fig. 5e	+>Kir2.1	w; +/UAS-Kir2.1::EGFP; +
	SDGs>Kir2.1	w; R21F01-p65.AD (attP40)/UAS-Kir2.1::EGFP; +/R93B07-GAL4.DBD (attP2)
Fig. 5g	ppk>ChR2, RGECO	w; +/13XLexAop2-IVS-NES-jRGECO1a-p10 (su(Hw)attP5); +/ppk-nlsLexA::p65 (attP2), 13XLexAop2-ChR2.T159C-HA (VK00013)
Fig. 5h	ppk>ChR2, RGECO; SDGs>Kir2.1	w; R21F01-p65.AD (attP40), UAS-Kir2.1::EGFP/13XLexAop2-IVS-NES-jRGECO1a-p10 (su(Hw)attP5); R93B07-GAL4.DBD (attP2)/ppk-nlsLexA::p65 (attP2), 13XLexAop2-ChR2.T159C-HA (VK00013)
Fig. 6a		w^{1118}; +; +
Fig. 6b		w^{1118}; +; +
Fig. 6c	+>Kir2.1	w; +/UAS-Kir2.1::EGFP; +

	CN>Kir2.1	*w*; *+/UAS-Kir2.1::EGFP*; *+/CN-GAL4*
Fig. 6d	*CN>GtACR1*	*w; +; UAS-GtACR1::EYFP* (attP2)*/CN-GAL4*
Fig. 6e	*+>NaChBac*	*w; +/UAS-NaChBac::EGFP; +*
	CN>+	*w; +; +/CN-GAL4*
	CN>NaChBac	*w; +/UAS-NaChBac::EGFP; +/CN-GAL4*
Fig. 6g	*CN>+*	*w; +; +/CN-GAL4*
	CN>CsChrimson	*w; +/20XUAS-CsChrimson::mCherry* (su(Hw)attP5)*; +/CN-GAL4*
Fig. 6h	*ppk>ChR2*, *RGECO*; *CN>+*	*w*; *+/13XLexAop2-IVS-NES-jRGECO1a-p10* (su(Hw)attP5); *CN-GAL4/ppk-nlsLexA::p65* (attP2), *13XLexAop2-ChR2.T159C-HA* (VK00013)
	ppk>ChR2, *RGECO*; *CN>CsChrimson*	*w*; *20XUAS-CsChrimson::mCherry* (su(Hw)attP5)*/13XLexAop2-IVS-NES-jRGECO1a-p10* (su(Hw)attP5); *CN-GAL4/ppk-nlsLexA::p65* (attP2), *13XLexAop2-ChR2.T159C-HA* (VK00013)
Supplementary Fig. 4a		w^{1118}; +; +
Supplementary Fig. 4b		w^{1118}; +; +
Supplementary Fig. 4c		w^{1118}; +; +
Supplementary Fig. 4d		w^{1118}; +; +
Supplementary Fig. 4e	*CN>CaLexA*	*w*; *LexAop-CD8-GFP-2A-CD8-GFP/+; UAS-mLexA-VP16-NFAT, LexAop-rCD2-GFP/CN-GAL4*
Fig. 7a	*+>Kir2.1*	*w*; *+/UAS-Kir2.1::EGFP*; +
	CN>Kir2.1	*w*; *+/UAS-Kir2.1::EGFP*; *+/CN-GAL4*
Fig. 7b	*+>*shi^{ts}	*w*; +; *+/UAS-*$Shibire^{ts}$
	Ilp2>shi^{ts}	*w*; *+/Ilp2-GAL4*; *+/UAS-*$Shibire^{ts}$
Fig. 7c	*+>*InR^{DN}	*w*; *+/UAS-InR.K1409A*; +
	SDGs>InR^{DN}	*w*; *R21F01-p65.AD* (attP40)*/UAS-InR.K1409A*; *R93B07-GAL4.DBD* (attP2)/+
Fig. 7d	*+>*InR^{CA}	*w*; *+/UAS-InR.Del*; +

	SDGs>InR^{CA}	*w*; *R21F01-p65.AD* (attP40)/*UAS-InR.Del*; *R93B07-GAL4.DBD* (attP2)/+
Fig. 7e	*SDGs>GCaMP*, *CsChrimson::mCherry*; *+>InR*^{DN}	*w*; *R21F01-p65.AD* (attP40), *20XUAS-IVS-jGCaMP7s* (su(Hw)attP5)/+; *R93B07-GAL4.DBD* (attP2)/*20XUAS-IVS-CsChrimson::mCherry* (VK00005)
	SDGs>GCaMP, *CsChrimson::mCherry*; *SDGs>InR*^{DN}	*w*; *R21F01-p65.AD* (attP40), *20XUAS-IVS-jGCaMP7s* (su(Hw)attP5)/*UAS-InR.K1409A*; *R93B07-GAL4.DBD* (attP2)/*20XUAS-IVS-CsChrimson::mCherry* (VK00005)
Fig. 7f	*SDGs>GCaMP*, *CsChrimson::mCherry*; *+>InR*^{CA}	*w*; *R21F01-p65.AD* (attP40), *20XUAS-IVS-jGCaMP7s* (su(Hw)attP5)/+; *R93B07-GAL4.DBD* (attP2)/*20XUAS-IVS-CsChrimson::mCherry* (VK00005)
	SDGs>GCaMP, CsChrimson::mCherry; *SDGs>InR*^{CA}	*w; R21F01-p65.AD* (attP40)*, 20XUAS-IVS-jGCaMP7s* (su(Hw)attP5)*/UAS-InR.Del; R93B07-GAL4.DBD* (attP2)*/20XUAS-IVS-CsChrimson::mCherry* (VK00005)
Supplementary Fig. 5a	*+>InR*^{DN}	*w; +/UAS-InR.K1409A; +*
	SDGs>InR^{DN}	*w; R21F01-p65.AD* (attP40)*/UAS-InR.K1409A; R93B07-GAL4.DBD* (attP2)/+
Supplementary Fig. 5b	*+>InR*^{DN}*, tub-GAL80*^{ts}	*w; +/UAS-InR.K1409A; +/tub-GAL80*^{ts}
	R16C06>InR^{DN}*, tub-GAL80*^{ts}	*w; +/UAS-InR.K1409A; R16C06-GAL4* (attP2)*/tub-GAL80*^{ts}
Supplementary Fig. 5c	*+>InR*^{CA}*, tub-GAL80*^{ts}	*w; +/UAS-InR.Del; +/ tub-GAL80*^{ts}
	R16C06>InR^{CA}*, tub-GAL80*^{ts}	*w; +/UAS-InR.Del; R16C06-GAL4* (attP2)*/tub-GAL80*^{ts}

Supplementary Table 2. Detailed conditions for Ca^{2+} imaging experiments

Imaging	Fig.4a	Fig.4b	Fig.4d	Fig.4e	Fig.4f	Fig.4g	Fig.5g	Fig.5h	Fig.6h	Fig.7e	Fig.7f
Laser system	Sapphire LP 488/561 (Coherent)	LDI-PRIME (Chroma Technology)	Sapphire LP 488/561 (Coherent)	LDI-PRIME (Chroma Technology)	LDI-PRIME (Chroma Technology)	VisiTech	LDI-PRIME (Chroma Technology)	LDI-PRIME (Chroma Technology)	LDI-PRIME (Chroma Technology)	LDI-PRIME (Chroma Technology)	LDI-PRIME (Chroma Technology)
Laser wavelength (nm)	561	561	561	561	561	561	561	561	561	561, 488	561, 488
Gain	Gain 1x	Gain 2x	Gain 3x	Gain 2x	Gain 2x	Gain 3x	Gain 2x	Gain 2x	Gain 2x	Gain 2x	Gain 2x
Digitizer (MHz)	10	10	10	10	10	10	10	10	10	10	10
EM Gain	150	100	50	100	100	500	100	100	100	100	200
Exposure time (ms)	100	200	500	200	200	800	200	200	200	200	500

Stimulation	Fig.4a	Fig.4b	Fig.4d	Fig.4e	Fig.4f	Fig.4g	Fig.5g	Fig.5h	Fig.6h
Wavelength (nm)	475	475	475	475	475	475	475	475	475
Duration (s)	15	15	1	3	3	60	3	3	3
Intensity ($\mu W/mm^2$)	6.24	6.24	6.24	6.24	6.24	208	6.24	4.16	6.24
