# Supplementary material for: Descending GABAergic pathway links brain sugar-sensing to peripheral nociceptive gating in Drosophila
Source: Nat Commun. 2023 Oct 16;14:6515. doi: 10.1038/s41467-023-42202-9 (PMC10579361; doi:10.1038/s41467-023-42202-9)
Supplement: Supplementary file 3 — Description of Additional Supplementary Files [file 41467_2023_42202_MOESM3_ESM.pdf]

Description of Additional Supplementary Files

File Name: Supplementary Movie 1
Description: Optogenetic stimulation of C4da neurons with or without *UAS-Kir2.1* driven by *SDGs-spGAL4*, related to Fig. 2a.

File Name: Supplementary Movie 2
Description: Mechanical stimulation of SDGs-silenced larvae, related to Fig. 2d.

File Name: Supplementary Movie 3
Description: Mechanical stimulation of larvae with sustained responsiveness of SDGs achieved by NaChBac expression, related to Fig. 2f.
