# Supplementary material for: Descending GABAergic pathway links brain sugar-sensing to peripheral nociceptive gating in Drosophila
Source: Nat Commun. 2023 Oct 16;14:6515. doi: 10.1038/s41467-023-42202-9 (PMC10579361; doi:10.1038/s41467-023-42202-9)
Supplement: Supplementary file 7 — Reporting Summary [file 41467_2023_42202_MOESM7_ESM.pdf]

nature portfolio

Corresponding author(s): Kazuo Emoto

Last updated by author(s): 14/9/23

Reporting Summary

Nature Portfolio wishes to improve the reproducibility of the work that we publish. This form provides structure for consistency and transparency in reporting. For further information on Nature Portfolio policies, see our Editorial Policies and the Editorial Policy Checklist.

Statistics

For all statistical analyses, confirm that the following items are present in the figure legend, table legend, main text, or Methods section.

n/a	Confirmed	
☐	☒	The exact sample size (*n*) for each experimental group/condition, given as a discrete number and unit of measurement
☐	☒	A statement on whether measurements were taken from distinct samples or whether the same sample was measured repeatedly
☐	☒	The statistical test(s) used AND whether they are one- or two-sided<br>*Only common tests should be described solely by name; describe more complex techniques in the Methods section.*
☐	☒	A description of all covariates tested
☐	☒	A description of any assumptions or corrections, such as tests of normality and adjustment for multiple comparisons
☐	☒	A full description of the statistical parameters including central tendency (e.g. means) or other basic estimates (e.g. regression coefficient) AND variation (e.g. standard deviation) or associated estimates of uncertainty (e.g. confidence intervals)
☐	☒	For null hypothesis testing, the test statistic (e.g. *F*, *t*, *r*) with confidence intervals, effect sizes, degrees of freedom and *P* value noted<br>*Give P values as exact values whenever suitable.*
☒	☐	For Bayesian analysis, information on the choice of priors and Markov chain Monte Carlo settings
☒	☐	For hierarchical and complex designs, identification of the appropriate level for tests and full reporting of outcomes
☒	☐	Estimates of effect sizes (e.g. Cohen's *d*, Pearson's *r*), indicating how they were calculated

Our web collection on statistics for biologists contains articles on many of the points above.

Software and code

Policy information about availability of computer code

Data collection	LASX (v1.1.0.12420), MetaMorph Offline (v77.5.0), MetaMorph (v7.10.4.407)
Data analysis	ImageJ (v1.53a), StackReg/TurboReg plugins of imageJ (available at http://bigwww.epfl.ch/thevenaz/stackreg/) for calcium imaging, Graphpad Prism (9.4.1), Microsoft Excel (v16.69.1), and VLC media player (v3.0.18 Vetinari) with an extension plugin "Time v3.2" (available at https://addons.videolan.org/p/1154032/), Adobe illustrator (27.6.1).

For manuscripts utilizing custom algorithms or software that are central to the research but not yet described in published literature, software must be made available to editors and reviewers. We strongly encourage code deposition in a community repository (e.g. GitHub). See the Nature Portfolio guidelines for submitting code & software for further information.

Data

Policy information about availability of data

All manuscripts must include a data availability statement. This statement should provide the following information, where applicable:

- Accession codes, unique identifiers, or web links for publicly available datasets
- A description of any restrictions on data availability
- For clinical datasets or third party data, please ensure that the statement adheres to our policy

Source data are provided with this paper as a Source Data file. Original movies used for data analysis are available from the corresponding author upon reasonable request.

Research involving human participants, their data, or biological material

Policy information about studies with human participants or human data. See also policy information about sex, gender (identity/presentation), and sexual orientation and race, ethnicity and racism.

Reporting on sex and gender	Not Applicable
Reporting on race, ethnicity, or other socially relevant groupings	Not Applicable
Population characteristics	Not Applicable
Recruitment	Not Applicable
Ethics oversight	not applicable

Note that full information on the approval of the study protocol must also be provided in the manuscript.

Field-specific reporting

Please select the one below that is the best fit for your research. If you are not sure, read the appropriate sections before making your selection.

☒ Life sciences ☐ Behavioural & social sciences ☐ Ecological, evolutionary & environmental sciences

For a reference copy of the document with all sections, see nature.com/documents/nr-reporting-summary-flat.pdf

Life sciences study design

All studies must disclose on these points even when the disclosure is negative.

Sample size	Sample sizes are provided in each figure. Power analyses were performed for behavioral experiments to determine the sample sizes that give a power (= 1 – β error probability) of >0.8, according to a previous report (Banerjee A, et al. 2009). For other experiments, sample sizes were chosen based on conventional standards used in our field. These values were determined based on the expected magnitude of inter-individual variability, given published results and our own data.
Data exclusions	No data were intensionally excluded.
Replication	Number of biological replicates are shown within each figure.
Randomization	Drosophila larvae of each genotype were randomly sampled from the population prepared for experiments. Covariates were not relevant in this study as experimental and control experiments were performed in parallel, and flies were maintained under identical rearing conditions. Flies were never arbitrarily assigned to treatment groups, and hence there were no experiments in which randomization could have been performed.
Blinding	Blinding was not relevant to this study because experimental flies were generated by and tested by the same investigator during data collection.

Reporting for specific materials, systems and methods

We require information from authors about some types of materials, experimental systems and methods used in many studies. Here, indicate whether each material, system or method listed is relevant to your study. If you are not sure if a list item applies to your research, read the appropriate section before selecting a response.

Materials & experimental systems

n/a	Involved in the study	
☐	☒	Antibodies
☒	☐	Eukaryotic cell lines
☒	☐	Palaeontology and archaeology
☐	☒	Animals and other organisms
☒	☐	Clinical data
☒	☐	Dual use research of concern
☒	☐	Plants

Methods

n/a	Involved in the study	
☒	☐	ChIP-seq
☒	☐	Flow cytometry
☒	☐	MRI-based neuroimaging

Antibodies

Antibodies used	Mouse anti-GFP (3E6) (1:200, clone 3E6, Thermo Fisher Scientific, #A11120),<br>Mouse anti-ChAT (4B1) (1:50, clone 4B1, Developmental Studies Hybridoma Bank)<br>Rabbit anti-GABA (1:100, Sigma-Aldrich, #A2052)<br>Rabbit anti-VGluT (1:400, A gift from H. Aberle (Mahr and Aberle, 2006))<br>Rabbit anti-Ilp2 (1:2000, A gift from T. Nishimura (Okamoto et al., 2012))<br>Goat anti-rabbit IgG (H+L) Alexa Fluor 633 (1:500, Thermo Fisher Scientific, #A21071)<br>Goat anti-mouse IgG (H+L) Alexa Fluor 635 (1:500, Thermo Fisher Scientific, #A31574)
Validation	All antibodies were validated by manufacturers and by other groups in the literatures, as described below:<br>Mouse anti-GFP (3E6): https://www.thermofisher.com/antibody/product/GFP-Antibody-clone-3E6-Monoclonal/A-11120<br>Mouse anti-ChAT (4B1): https://dshb.biology.uiowa.edu/ChAT4B1<br>Rabbit anti-GABA: https://www.sigmaaldrich.com/US/en/product/sigma/a2052<br>Rabbit anti-VGluT: validated by Mahr and Aberle, 2006.<br>Rabbit anti-Ilp2: validated by Okamoto et al., 2012.<br>Goat anti-rabbit IgG (H+L) Alexa Fluor 633: https://www.thermofisher.com/antibody/product/Goat-anti-Rabbit-IgG-H-L-Highly-Cross-Adsorbed-Secondary-Antibody-Polyclonal/A-21071<br>Goat anti-mouse IgG (H+L) Alexa Fluor 635: https://www.thermofisher.com/antibody/product/Goat-anti-Mouse-IgG-H-L-Cross-Adsorbed-Secondary-Antibody-Polyclonal/A-31574

Animals and other research organisms

Policy information about studies involving animals; ARRIVE guidelines recommended for reporting animal research, and Sex and Gender in Research

Laboratory animals	Drosophila melanogaster w[1118] was used as the control strain.<br>The following fly strains were obtained from Bloomington Drosophila Stock Center: UAS-CD4-tdTomato in VK00033, UAS-Stinger (BL#90914), ppk-CD4-tdTomato on 2nd chromosome (BL#35844), ppk-CD4-tdTomato on 3rd chromosome (BL#35845), 10XUAS-mCD8::GFP in attP2 (BL#32184), 20XUAS-IVS-mCD8::GFP in attP2 (BL#32194), 20XUAS-CsChrimson::mCherry in su(Hw)attP5 (BL#82181), UAS-nsyb-spGFP1-10, LexAop-CD4-spGFP11 (BL#64314), R16C06-GAL4 in attP2 (BL#48719), R21F01-p65.AD in attP40, R93B07-GAL4.DBD in attP2 (BL#69254), 10XUAS-mCD8::RFP in attP18; 13XLexAop2-mCD8::GFP in su(Hw)attP8 (BL#32229), UAS-Kir2.1::EGFP (BL#6596), LexAop-ReaChR in VK00005 (BL#53746), UAS-GtACR1::EYFP in attP2 (BL#92983), UAS-NaChBac::EGFP (BL#9466), 13XLexAop-IVS-jGCaMP7s in VK00005 (BL#80913), Gad1-Trojan-LexA-QFAD (BL#60324), CaryP in attP40 (BL#36304), UAS-IR-Gad1 (TRiP.HMC03350) in attP40 (BL#51794), UAS-IR-Gad1 (TRiP.JF02916) in attP2 (BL#28079), UAS-Dcr-2 on 2nd (BL#24650) or 3rd (BL#24650) chromosome, GABAB-R12A-GAL4 (BL#84701), GABAB-R22A-GAL4 (BL#84634), CaryP in attP2 (BL#36303), UAS-IR-GABAB-R1 (TRiP.HMC03388) in attP2 (BL#51817), UAS-IR-GABAB-R2 (TRiP.HMC02975) in attP2 (BL#50608), GABAB-R32A-AD-GAL4 (BL#84635), Rdl2A-GAL4 (BL#84688), UAS-IR-GABAB-R3 (TRiP.HMC02989) in attP40 (BL#50622), UAS-IR-Rdl (TRiP.HMC03643) in attP40 (BL#52903), 13XLexAop2-IVS-NES-jRGECO1a-p10 in su(Hw)attP5 (BL#64426), 13XLexAop2-ChR2.T159C-HA in VK00013 (BL#52256), 20XUAS-ChR2.T159C-HA in VK00018 (BL#52258), ilp2-GAL4 (BL#37516), UAS-InR.K1409A as InRDN (BL#8252), UAS-InR.Del as InRCA (BL#8248), tub-GAL80ts (BL#7017), 20XUAS-IVS-jGCaMP7s in su(Hw)attP5 (BL#80905), 20XUAS-IVS-CsChrimson::mCherry in VK00005 (BL#82180), and the CaLexA reporter (LexAop-CD8-GFP-2A-CD8-GFP; UAS-mLexA-VP16-NFAT, LexAop-rCD2-GFP) (BL#66542). The following RNAi lines were obtained from Vienna Drosophila Resource Center: UAS-IR-Gad1 (GD8508 on 2nd chromosome, BL#32344), UAS-IR-GABAB-R1 (KK109166) in VIE260b (VDRC#101440), UAS-IR-GABAB-R2 (KK100020) in VIE260b (VDRC#110268). ppk-nlsLexA::p65 in attP2, ppk-GAL4 on X chromosome, and 20XUAS-droRGECO were generated in our lab; UAS-brpD3::mCherry was from T. Suzuki; UAS-PTX was from G. Roman (Ferris et. al., 2006); CN-GAL4 (VT58471-GAL4, Cha-GAL80) was from G. S. B. Suh (Oh et. al., 2019); UAS-shibirets was from T. Kitamoto (Murphey et. al., 2003). R16C06-LexA was created by cloning the enhancer region of R16C06-GAL4 into the pBPLexA::p65Uw vector (Addgene #26231), followed by phiC31-mediated transgenesis targeting the attP40 insertion site (BestGene Inc.).
Wild animals	The present study did not utilize wild animals.
Reporting on sex	Both male and female fly larvae were used without any intensional selection, except for experiments using larvae with a GAL4 driver inserted in the X chromosome. In these experiments, varied expression from the GAL4/UAS system may occur due to the X-chromosome dosage compensation if both sexes were mixed. To avoid this issue, we selected male larvae only for these experiments.
Field-collected samples	The present study did not utilize field-collected samples.
Ethics oversight	Animal experiments using transgenic flies were conducted with the approval of Research Ethics Committee of the University of Tokyo.

Note that full information on the approval of the study protocol must also be provided in the manuscript.
