# Supplementary material for: Descending GABAergic pathway links brain sugar-sensing to peripheral nociceptive gating in Drosophila
Source: Nat Commun. 2023 Oct 16;14:6515. doi: 10.1038/s41467-023-42202-9 (PMC10579361; doi:10.1038/s41467-023-42202-9)
Supplement: Supplementary file 9 — Editor summary OLD [file 41467_2023_42202_MOESM9_ESM.docx]

Escape behavior is dynamically altered by animals' physiological conditions. Here, the authors identify in Drosophila larvae a cluster of GABAergic descending neurons that mediate nociceptive modulation upon nutritional changes.
